# Editorial: Mechanical coupling between extracellular and intracellular microenvironment

**DOI:** 10.3389/fcell.2024.1427439

**Published:** 2024-05-20

**Authors:** Saumendra Kumar Bajpai, Yongho Bae, Sreenath Balakrishnan, Dong-Hwee Kim

**Affiliations:** ^1^ Indian Institute of Technology Madras, Chennai, India; ^2^ Department of Pathology and Anatomical Sciences, University at Buffalo, Buffalo, NY, United States; ^3^ Indian Institute of Technology Goa, Ponda, India; ^4^ Department of Integrative Energy Engineering, Korea University, Seoul, India

**Keywords:** cell differentiation, extracellular matrix, cell stretch, mechanotranscription, mechanotransducer

While inviting contributions on this Research Topic, our aim was to receive research articles that highlight the spatiotemporal mechanical heterogeneity of cells, particularly those correlating with various pathological conditions. From the handful of such contributions we received, we perceive the cell as being an extremely redundant system, capable of responding to a wide variety of cues simultaneously. Whether this extreme redundancy represents an evolutionary solution to the challenges of life remains a moot point.

Even more immediate is the realization that just as mesenchymal stem cells can differentiate under almost any perturbation in physicochemical environment, such as via substrate-strain, as reported by Lee et al. mandibular fibrochondrocytes can undergo drastic changes in phenotype (Ahn et al.) with mere depletion of serum in culture medium. A change in cell-migration or phenotype differentiation exemplifies the extensive redundancy of responses exhibited by a cell in response to changes in receptor-ligand binding or receptor-force interactions. For both types of responses, there are probably as many examples as there are identified receptors on the plasma membrane.

The net picture of the cell that emerges from the reports presented in this collection, as well as similar works elsewhere, is that of an infinite-equilibrium system capable of inter-state transitions using multiple intermediate quasi-equilibrium pathways. While this picture of cell-dynamics begs examination via principles of stability and bifurcation in non-linear dynamical systems, such analysis will likely follow further exploration of pathways that allow such a dynamical and redundant transition in cellular phenotype, in response to changes in the mechanochemical environment. A redundancy-adjusted non-linear dynamical model of the cell could be, in the long run, the holy grail of theranostics.

